# Multichromosomal median and halving problems under different genomic distances

**DOI:** 10.1186/1471-2105-10-120

**Published:** 2009-04-22

**Authors:** Eric Tannier, Chunfang Zheng, David Sankoff

**Affiliations:** 1 INRIA Rhône-Alpes, Inovallée, 655 avenue de l'Europe, Montbonnot, 38 334 Saint Ismier Cedex, France; 2 Université de Lyon, F-69000, Lyon, Université Lyon 1, CNRS, UMR5558, Laboratoire de Biométrie et Biologie Évolutive, F-69622, Villeurbanne, France; 3 Department of Biology and Department of Mathematics and Statistics, University of Ottawa, Ottawa, Canada K1N 6N5

## Abstract

**Background:**

Genome median and genome halving are combinatorial optimization problems that aim at reconstructing ancestral genomes as well as the evolutionary events leading from the ancestor to extant species. Exploring complexity issues is a first step towards devising efficient algorithms. The complexity of the median problem for unichromosomal genomes (permutations) has been settled for both the breakpoint distance and the reversal distance. Although the multichromosomal case has often been assumed to be a simple generalization of the unichromosomal case, it is also a relaxation so that complexity in this context does not follow from existing results, and is open for all distances.

**Results:**

We settle here the complexity of several genome median and halving problems, including a surprising polynomial result for the breakpoint median and guided halving problems in genomes with circular and linear chromosomes, showing that the multichromosomal problem is actually easier than the unichromosomal problem. Still other variants of these problems are NP-complete, including the DCJ double distance problem, previously mentioned as an open question. We list the remaining open problems.

**Conclusion:**

This theoretical study clears up a wide swathe of the algorithmical study of genome rearrangements with multiple multichromosomal genomes.

## Background

The gene order or syntenic arrangement of ancestral genomes may be reconstructed based on comparative evidence from present-day genomes – the phylogenetic approach – or on internal evidence in the case of genomes descended from an ancestral polyploidisation event, or from a combination of the two. The computational problem at the heart of phylogenetic analysis is the *median problem*, while internal reconstruction inspires the *halving problem*, and the combined approach gives rise to *guided halving*. How these problems are formulated depends (1) on the karyotypic framework: the number of chromosomes in a genome and whether they are constrained to be linear, or if circular chromosomes are also permitted, and (2) on the objective function used to evaluate possible solutions. This function is based on some notion of genomic distance, either the number of adjacent elements on a chromosome in one genome that are disrupted in another – the breakpoint distance – or the number of evolutionary operations necessary to transform one genome to another.

While the karyotypes allowed in an ancestor vary only according to the dimensions of single versus multiple chromosome, and linear versus circular versus mixed, the genomic distances of interest have proliferated according to the kinds of evolutionary operations considered, from the classic, relatively constrained, reversals/translocations distance to the more inclusive *Double Cut-and-Join *(DCJ) measure, and many others [1].

The computational complexity of some of these problems has been settled for some specific distances and karyotypic contexts, and it is sometimes taken for granted that these results carry over to other combinations of context and distance. This is not necessarily the case. In this paper, we survey the known results and unsolved cases for three distance measures in three kinds of karyotype. We include several results presented here for the first time, as well as discussions on the definitions of the distances. The results contain both new polynomial-time algorithms and NP-hardness proofs. This paper is the full version of an extended abstract that has appeared in [2], which announced the results without giving all the proofs. In particular, a full discussion on the breakpoint distance definition, as well as the proofs of Theorem 2, Theorem 4, and Theorem 6 are added here, which makes this version a complete and definitive one.

### Genomes, breakpoints and rearrangements

#### Multichromosomal genomes

**Figure 1:**
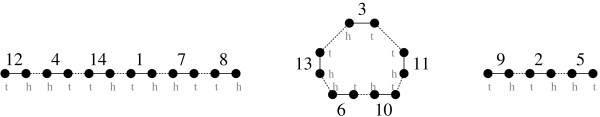
**The graph *GΠ*_Π _of a genome Π**. Π is a genome on the set of genes {1,...,14}, containing three chromosomes, two of them being linear and one circular. Its adjacencies are the union of *C*_1 _= {12^*h*^4^*h*^, 4^*t*^14^*t*^, 14^*h*^1^*t*^, 1^*h*^7^*h*^, 7^*t*^8^*t*^}, *C*2 = {3^*t*^11^*t*^, 11^*h*^10^*t*^, 10^*h*^6^*t*^, 6^*h*^13^*h*^, 13^*t*^3^*h*^} and *C*_3 _= {9^*h*^2^*t*^, 2^*h*^5^*h*^}. It has four telomeres.

We follow the general formulation of a genome in [3]. A *gene A *is an oriented sequence of DNA, identified by its *tail A*^*t *^and its *head A*^*h*^. Tails and heads are the *extremities *of the genes. An *adjacency *is an unordered pair of gene extremities. A *genome *Π is a set of adjacencies on a set of genes. Each adjacency in a genome means that two gene extremities are consecutive on the DNA molecule. In a genome, each gene extremity is adjacent to zero or one other extremity. An extremity *x *that is not adjacent to any other extremity is called a *telomere*, and can be written as an adjacency *x*∘ with a null symbol ∘. The adjacency *x*∘ is called a *telomeric adjacency*. For a genome Π on a set of genes , consider the graph *G*_Π _whose vertices are all the extremities of the genes, and the edges include all the non telomeric adjacencies in Π as well as an edge joining the head and the tail of each gene. This graph is a set of disjoint paths and cycles. Every connected component is called a *chromosome *of Π. A chromosome is *linear *if it is a path, and *circular *if it is a cycle. A genome with only linear, or only circular, chromosomes is called a *linear *or *circular *genome, respectively. An example of a graph *G*_Π _is given in Figure 1.

A Genome can also be represented as a set of strings, by writing the genes for each chromosome in the order in which they appear in the paths and cycles of the graph *G*_Π_, with a bar over the gene if the head of the gene appears before the tail (we say it has *negative *sign), and none if the tail appears before the head (it has *positive *sign). For each linear chromosome, there are two possible equivalent strings, according to the arbitrary chosen starting point. One is obtained from the other by reversing the order and switching the signs of all the genes. For circular chromosomes, there are also two possible circular string representations, according to the direction in which the cycle is traversed. For example, chromosome *C*_1 _of the genome Π of Figure 1 may be written (12  14 1  8) or ( 7  4 ).

A genome with only one chromosome is called *unichromosomal*. These correspond to *signed permutations*: the two string representations are (linear or circular) signed permutations.

#### Genomes with duplicates

A *duplicated gene A *is a couple of homologous oriented sequences of DNA, identified by two tails *A*1^*t *^and *A*2^*t*^, and two heads *A*1^*h *^and *A*2^*h*^. An *all-duplicates genome Δ *is a set of adjacencies on a set of duplicated genes.

For a genome Π on a gene set , a *doubled genome *Π ⊕ Π is an all-duplicates genome on the set of duplicated genes from  such that if *A*^*x*^*B*^*y *^(*x, y *∈ {*t, h*}) is an (possibly telomeric) adjacency of Π (*A*^*x *^or *B*^*y *^may be ∘), either *A*1^*x*^*B*1^*y *^and *A*2^*x*^*B*2^*y*^, or *A*2^*x*^*B*1^*y *^and *A*1^*x*^*B*2^*y*^, are adjacencies of Π ⊕ Π.

**Figure 2:**
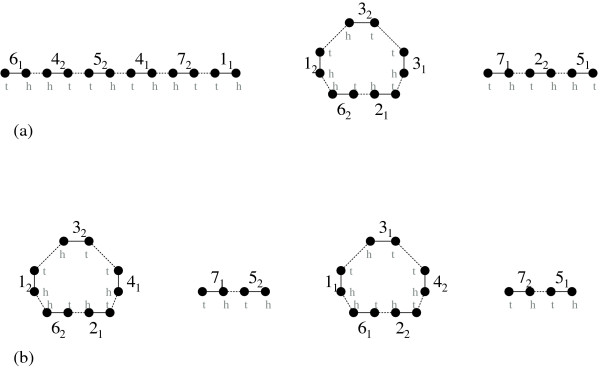
**The graphs and *G*_Δ _*G*_Π⊕Π _of an all-duplicates genome Δ and a doubled genome Π ⊕ Π**. (a) Δ has three chromosomes, while (b) Π ⊕ Π has four, and consist of two copies of two chromosomes, that have the same set of duplicated genes {1,...,7}.

Note the difference between a general all-duplicates genome and the special case of a doubled genome: the former has two copies of each gene, while in the latter these copies are organised in such a way that there are two identical copies of each chromosome when we ignore the 1's and 2's in the *A*1^*x*^'s and *A*2^*x*^'s: it has two linear copies of each linear chromosome, and for each circular chromosome, either two circular copies or one circular chromosome containing the two successive copiesNote also that for a genome Π, there is an exponential number of possible doubled genomes Π ⊕ Π (exactly two to the power of the number of non-telomeric adjacencies in Π). These definitions correspond to duplicated and perfectly duplicated genomes found in [4], and slightly differs from the perfectly duplicated genome definition found in [5], as discussed in [4]. An example of an all-duplicates genome and a doubled genome is shown in Figure 2. Doubled genomes are the immediate result of an evolutionary event called *Whole Genome Duplication *(WGD), which is known to have occurred in many evolutionary lineages, from protists [6] to yeasts, to plants, to fish, to amphibians and even to mammals [7]. All-duplicates genomes derive from doubled genomes through a series of rearrangement events. Typically, all-duplicates genomes pertain to extant species, while doubled genomes are ancestral configurations inferred to exist immediately after the WGD, and that are to be reconstructed.

In discussing all-duplicates genomes, we will sometimes contrast them with *ordinary genomes *which have a single copy of each gene.

#### The breakpoint distance

The breakpoint distance has been well-studied for permutations, i.e., unichromosomal genomes [8,9], but only a few published discussions have focused on how it should be defined for multichromosomal genomes (see [10] for one suggestion). The distance should depend not only on common adjacencies, or rather their absence, but also on common telomeres (or lack thereof) in two genomes. Here we propose a definition that we wish valid for all types of karyotypes, based on a most general approach integrating all possible informations from the two genomes. For two genomes Π and Γ on a set  of *n *genes, suppose Π has *N*_Π _chromosomes, and Γ has *N*_Γ _chromosomes. Let *a*(Π, Γ) be the number of common adjacencies, *e*(Π, Γ) be the number of common telomeres of Π and Γ. Then insofar as it should depend additively on these components, we may suppose the breakpoint distance has form



where *β*, *θ *and *γ *are positive parameters, while *ψ *may have either sign. Taking Π = Γ and imposing *d*_*BP *_(Π, Π) = 0 yields the relations *β *= 1 and 1 - 2*θ *+ 2*γ *= 0, so *θ *= *γ *+ 1/2, and the distance formula reduces to:



It is most plausible to count a total of 1 breakpoint for a fusion or fussion of linear chromosomes, which implies *γ *= *ψ *= 0, so the most natural choice of *breakpoint distance *between Π and Γ is



It might be argued that a fussion or fusion should count for as many as 2 breakpoints, or anything between 1 and 2, so that alternate values of *γ *and *ψ *might be entertained, provided *γ *∈ [0, ], and *ψ *∈ [0,1 - *γ*]. This may have an influence on how to calculate the number of breakages within a scenario, as discussed in [11]. For example, the parameters chosen in [10] are *γ *=  and *ψ *= , giving rise to the disadvantage of there possibly being more breakpoints between two genomes than adjacencies in either one. For example, in comparing Π = (1 2 3 4 5) and Γ in which five linear chromosomes each contain one gene *i *∈ {1,...,5}, the definition in [10] would count 9 breakpoints, which seems counterintuitive, while our definition counts 4, which seems more reasonable. Whether all the results presented in this paper also hold for the definition in [10] is open.

The definition of the breakpoint distance is easily transposable to the comparison of two all-duplicates genomes. For one all-duplicates genome Δ and one ordinary genome Π, the *breakpoint distance *between Π and Δ is the minimum breakpoint distance between Δ and a doubled genome Π ⊕ Π, that is,



#### The Double Cut-and-Join distance

**Figure 3:**
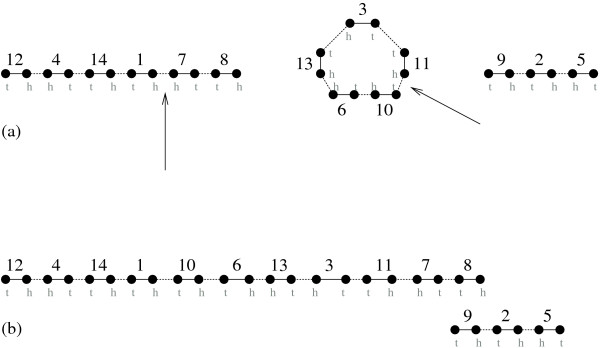
**A DCJ operation on the genome Π of Figure 1**. Adjacencies 1^*h*^7^*h *^and 10^*t*^11^*h *^of the genome represented in (a) are cut and adjacencies 1^*h*^10^*t *^and 11^*h*^7^*h *^are joined to construct the genome represented in (b). This example shows how a DCJ operation can fuse two chromosomes into one.

Given a genome Π, a double-cut-and-join (DCJ) is an operation *ρ *acting on two adjacencies *pq *and *rs *(possibly some of *p, q, r, s *are ∘ symbols, so that telomeric adjacencies are considered; one adjacency can even be ∘∘). The DCJ operation replaces *pq *and *rs *either by *pr *and *qs*, or *ps *and *qr*. An example of DCJ operation on the genome Π of Figure 1 is drawn in Figure 3.

A DCJ can reverse an interval of a genome, may cause the fussion of one chromosome into two, or the fusion of two chromosomes into a one, or a reciprocal translocation: the exchange of two telomere-containing segments between two chromosomes. Two consecutive DCJ operations, excising and circularising a chromosomal segment followed by a re-linearisation of the *circular intermediate *and reintegration on the same chromosome, using two new cut-points, results in a *block interchange*: two segments of the genome appear to simply exchange their positions. In the case these two segments are consecutive, the two DCJs result in a *transposition*, the apparent movement of a segment from one place on a chromosome to another. The DCJ operation is thus a very general framework, introduced by Yancopoulos *et al*. [12], as well as by Lin *et al*. in a special case [13], and since been adopted by Bergeron *et al*. [3,14] and many others, sometimes under other names such as spring [15] or "2-break rearrangement" [16].

If Π and Γ are two genomes on a set  of *n *genes, the minimum number of DCJ operations needed to transform Π into Γ is called the *DCJ distance *and noted *d*_*DCJ*_(Π, Γ).

This DCJ distance is easily defined also for two all-duplicates genomes. For one all-duplicates genome Δ and one ordinary genome Π, the *DCJ distance *between Π and Δ is *d*_*DCJ*_(Π, Δ) = min_Π⊕Π _*d*_*DCJ*_(Π ⊕ Π, Δ).

#### The reversal/translocation distance

The reversal/translocation distance was introduced by Hannenhalli and Pevzner [17], and is equivalent to the DCJ distance constrained to linear genomes.

If Π is a linear genome, a *linear *DCJ operation is a DCJ operation on Π that results in a linear genome. This allows reversals, chromosome fusions, fussions, and reciprocal translocations. DCJs that create circular intermediates, temporary circular chromosomes, and thereby mimic block interchanges and transpositions, are not allowed. Chromosome fusions and fussions are particular cases of translocations in this framework, justifying the appellation *RT-distance*. If Π and Γ are linear genomes, the *RT *distance between Π and Γ is the minimum number of linear DCJ operations that transform Π into Γ, and is noted *d*_*RT *_(Π, Γ).

### Computational problems

The classical literature on genome rearrangements aims at reconstructing the evolutionary events and ancestral configurations that explain the differences between the organization of extant genomes. The focus has been on the genomic distance, median and halving problems. More recently the doubled distance and guided halving problems have also emerged as important. In each of the ensuing sections of this paper, these five problems are examined for a specific combination of distance *d *(breakpoint, DCJ or RT) and kind of multichromosomal karyotype (linear, circular, mixed).

1. **Distance**. Given two genomes Π, Γ, compute *d*(Π, Γ). Once the distance is calculated, an additional problem in the cases of DCJ and RT is to reconstruct the rearrangement scenario of length *d*(Π, Γ), i.e. the putative events that differentiate the genomes.

2. **Double distance**. Given an all-duplicates genome Δ and an ordinary genome Π, compute *d*(Δ, Π). This computation evaluates the evolutionary distance posterior to a WGD of the given genome Π, leading to an all-duplicates genome Δ, and locates the genes of the all-duplicates genome on chromosomes in one of the two ancestral copies of the ordinary genome. Because the assignment of labels "1" or "2" to the two identical (for our purposes) copies of a duplicated gene in Δ is arbitrary, the double distance problem is equivalent to finding such an assignment that minimises the distance between Δ and a genome Π ⊕ Π considered as ordinary genomes, where all the genes on any one chromosome in Π ⊕ Π are uniformly labeled "1" or "2" [16,18]. The double distance function is not symmetric because Δ is an all-duplicates genome and Π is an ordinary one, thus capturing the presumed asymmetric temporal and evolutionary relationship between the ancestor Π and the present-day genome Δ.

3. **Median**. Given three genomes Π_1_, Π_2_, Π_3_, find a genome *M *which minimises *d*(Π_1_, *M*) + *d*(Π_2_, *M*) + *d*(Π_3_, *M*). The median problem estimates the common ancestor of two genomes, given a third one as an outgroup. This is meaningful even in the "unrooted" case, where it is not specified which of the three genomes is the outgroup, because of the symmetry of the sum to be minimised.

4. **Halving**. Given an all-duplicates genome Δ, find an ordinary genome Π which minimises *d*(Δ, Π), the double distance mentioned above. The goal of a halving analysis is to reconstruct the ancestor of an all-duplicates genome at the time of a WGD event.

5. **Guided halving**. Given an all-duplicates genome Δ and an ordinary genome Π, find an ordinary genome *M *which minimises *d*(Δ, *M*) + *d*(*M*, Π). The guided halving problem is similar to the genome halving problem for Δ, but it takes into account the ordinary genome Π of an organism presumed to share a common ancestor with *M*, the reconstructed undoubled ancestor of Δ. A variant of the guided halving problem introduced in [19] is to find an ordinary genome *M *that is a solution to genome halving, that is, minimises *d*(Δ, *M*), and which in addition minimises *d*(*M, Π*). This helps choosing, among the numerous solutions to the genome halving problem, the one that is closest to the outgroup. We do not study this variant here, and it is open for all genomic distances.

We will survey these five computational problems for the three distances that we have introduced, in the cases of multichromosomal genomes containing all linear chromosomes, all circular chromosomes, or permitting both. The latter are refered as *mixed genomes*.

While many problems are open for multichromosomal genomes, there is a huge amount of research on these problems for unichromosomal genomes, whether circular or linear (the two cases are often equivalent up to some transformations [1]). They are not systematically particular cases of the multichromosomal problems, as the constraint of keeping only one chromosome along a rearrangement scenario can result in more difficult problems. More precisely, unichromosomal DCJ problems reduce to RT multichromosomal ones. Indeed, the RT operations always transform a unichromosomal genome into a unichromosomal one. As this paper contains very few results on the RT distance, practically the unichromosomal cases are often independent and not generalized here. Results on unichromosomal genomes are summarised in Table 1, together with the results for the multichromosomal case we review or present here. A complete survey on these problems can be found in [1].

## Results

### Breakpoint distance, circular and mixed genomes

In this section, *d *= *d*_*BP*_, and genomes are considered in their most general definition, that is, multichromosomal with both circular and linear chromosomes allowed. All the results also stand for circular genomes, but not always for linear genomes, which will be considered in a following section. As the nuclear genome of a eukaryotic species, a mixed karyotype is rarely observed, so probably unstable. Nevertheless this case is of great theoretical interest, as it is the only combination of distance and karyotype where all five problems mentioned in the previous section prove to be polynomially solvable, including the median problem which is hard for almost every other variant. Furthermore, the solutions in this context may suggest approaches for other variants of the problems, as well as providing a rapid bound for other distances, through the Watterson *et al*. bound [8].

#### Distance and double distance

The distance computation follows directly from the definition, and is easily achievable in linear time. The double distance computation is also easy: let Π be a genome and Δ be an all-duplicates genome. Let *a*(Π, Δ) be the sum, for every adjacency *xy *in Π, of the number of adjacencies among *x*1*y*1, *x*1*y*2, *x*2*y*1, *x*2*y*2 in Δ. Let *e*(Π, Δ) be the sum, for every telomere *x *in Π, of the number of telomeres among *x*_1 _and *x*_2 _in Δ.

Then we obtain



Indeed, it is a lower bound on the distance, because *a*(Π, Δ) and *e*(Π, Δ) are upper bounds on the number of common adjacencies and common telomeres, respectively, between Δ and any Π ⊕ Π. This lower bound is attained by constructing Π ⊕ Π in the following way: let *xy *be a possibly telomeric adjacency in Π (either *x *or *y *may be ∘ symbols); if *x*1*y*1 or *x*2*y*2 is an adjacency in Δ, choose *x*1*y*1 and *x*2*y*2 as adjacencies in Π ⊕ Π; If *x*1*y*2 or *x*2*y*1 is an adjacency in Δ, choose *x*1*y*2 and *x*2*y*1 as adjacencies in Π ⊕ Π; the two cases are either mutually exclusive if *xy *is not telomeric, or identical if *xy *is telomeric, so the assignment is made without ambiguity. For all adjacencies that have not been assigned, assign them arbitrarily.

#### Median

The following result contrasts with the NP-completeness proofs of almost all median problems in the literature [20-22] (see [23,24] for tractability results on some variants). The problem is NP-complete for unichromosomal genomes, that is, when the median genome *M *is required to be unichromosomal, whether the genomes are linear or circular [20,21], but the multichromosomal case happens to be easier.

**Theorem 1**. *There is a polynomial time algorithm for the breakpoint median problem for multichromosomal genomes*.

*Proof*. Let Π_1_, Π_2_, Π_3 _be three genomes on a gene set  of size *n*. For any genome *M *on , let *s*(*M*) = *d*(Π_1_, *M*) + *d*(Π_2_, *M*) + *d*(Π_3_, *M*) be the *median score *of *M*.

Draw a graph *G *on the vertex set containing (1) all extremities of genes in , and (2) one supplementary vertex *t*_*x *_for every gene extremity *x*. For any pair of gene extremities *x, y*, draw an edge *xy *weighted by the number of genomes, among Π_1_, Π_2_, Π_3_, for which *xy *is an adjacency. Then there is an edge between each pair of gene extremities, weighted by 0, 1, 2, or 3. Now for any vertex *x*, draw an edge *xt*_*x *_weighted by half the number of genomes, among Π_1_, Π_2_, Π_3_, having *x *as a telomere. Each edge *xt*_*x *_is then weighted by 0, , 1, or . Finally, put an edge of weight 0 between *t*_*x *_and *t*_*y *_for all pairs of gene extremities *x, y*. Let *M *be a perfect matching in *G*. Clearly, the edges joining gene extremities in *M *define the adjacencies of a genome, which we also call *M*. The relation between the weight of the perfect matching *M *and the median score of the genome *M *is easy to state:

**Claim 1**. *The weight w*(*M*) *of the perfect matching M in G is *3*n *- *s*(*M*).

Indeed, for any genome Π_*i*_, , where *a*_*i *_= *a*(Π_*i*_, *M*) is the number of common adjacencies between *M *and Π_*i*_, and *e*_*i *_= *e*(Π_*i*_, *M*) is the number of common telomeres between *M *and Π_*i*_. If *M *and Π_*i *_have a common adjacency or a common telomere, this accounts for 1 or , respectively, in the weight of the perfect matching *M*. So the weight of the matching *M *is , which yields *d*(Π_1_, *M*) + *d*(Π_2_, *M*) + *d*(Π_3_, *M*) = 3*n *- *w*(*M*).

Conversely, any genome *M *can be extended to a perfect matching *M *in *G *such that *s*(*M*) = 3*n *- *w*(*M*): construct the matching *M *by including the edges *xy *and *t*_*x*_*t*_*y *_for each adjacency *xy *and an edge *xt*_*x *_for each telomere *x*.

Claim 1 implies that a maximum weight perfect matching *M *is a minimum score median genome. As the maximum weight perfect matching problem is polynomial [25], so is the breakpoint median problem.   □

If the three genomes in the instance are circular, then it is possible to constrain the result to also be circular by restricting the graph *G *to the extremities of the genes. Then, in the same way, a perfect matching gives a circular solution to the median problem. This is not the case for linear genomes, since there is no way to guaranty that no chromosome in an instance is circular.

Note that a generalisation of this algorithm remains valid if the median of more than three genomes is to be computed. The phylogeny problems, both "big" and "small" versions, which also generalise the median problem for three genomes, remain open. The big problem is the search for a Steiner tree in the space of genomes, minimising the sum of the distances on its branches, while in the small problem, presumably easier, the graph-theoretical structure of the tree, namely its vertex set and edge or branch set, are given, and only the genomes corresponding to the extra vertices (not corresponding to the given genomes) need to be reconstructed.

#### Halving

To our knowledge, the genome halving with breakpoint distance has not yet been studied. In this framework, it has an easy solution, using a combination of elements from the maximum weight perfect matching technique in the solution of the median problem presented above, and the double distance computation. Let Δ be an all-duplicates genome on a gene set , and *G *be the graph on the vertex set containing (1) all the extremities of the genes in , and (2) one supplementary vertex *t*_*x *_for every gene extremity *x*. For any pair of gene extremities *x, y*, draw an edge in *G *weighted by zero, one or two according to the number of adjacencies in Δ among *x*1*y*1, *x*1*y*2, *x*2*y*1, and *x*2*y*2. Now for any vertex *x*, draw an edge *xt*_*x *_weighted by half the number of telomeres among *x*1 and *x*2 in Δ. Finally, put an edge of weight 0 between *t*_*x*_*t*_*y *_for all pairs of gene extremities *x, y*.

For a genome *M *on , define a perfect matching, also called *M*, by including edges *xy *and *t*_*x*_*t*_*y *_for each adjacency *xy*, and an edge *xt*_*x *_for each telomere *x*. Let *w*(*M*) be the weight of the matching *M*.

**Claim 2**. *For a genome M on , the perfect matching M thus constructed satisfies w*(*M*) = 2*n *- *d*(Δ, *M*).

Indeed, the score of the perfect matching *M *is , that is, 2*n *- *d*(Δ,*M*), according to the double distance formula (see above in this section).

Conversely, it is easy to see that any perfect matching on *G *defines a genome *M *such that *w*(*M*) = 2*n *- *d*(Δ, *M*). This implies that the maximum weight perfect matching solves the genome halving problem in the breakpoint distance context.

Again, it is possible to solve the problem on only circular genomes by restricting the graph *G *to the gene extremities, dropping the *t*_*x *_supplementary vertices.

#### Guided Halving

As is the case for the median problem, this context provides the only polynomial result for the guided genome halving problem up to our knowledge. The solution combines elements of the three previous results, on the double distance, median and halving problems.

Let Δ be an all-duplicates genome on a gene set , and Π be an ordinary genome on . Let *G *be the graph on the vertex set containing (1) all the extremities of the genes in , and (2) one supplementary vertex *t*_*x *_for every gene extremity *x*.

For any pair of gene extremities *x, y*, there is an edge in *G *weighted by the number of adjacencies among *x*1*y*1, *x*1*y*2, *x*2*y*1, *x*2*y*2 in Δ, and *xy *in Π. Now there is an edge *xt*_*x *_for any gene extremity *x *weighted by half the number of telomeres among *x*1, *x*2 in Δ and *x *in Π. So each edge between gene extremities has an integer weight in {0, 1, 2, 3}, and *xt*_*x *_edges may have weight 0, , 1, or . Add 0-weight edges *t*_*x*_*t*_*y *_for all pairs *x, y *of gene extremities.

For any genome *M*, let *s*(*M*) = *d*(Δ, *M*) + *d*(*M*, Π). It is possible to construct a perfect matching *M *in *G *from genome *M *by choosing edges *xy *and *t*_*x*_*t*_*y *_for every adjacency *xy *in *M*. Its weight is denoted *w*(*M*).

**Claim 3**. *For a genome M, the perfect matching thus constructed satisfies w*(*M*) = 3*n *- *s*(*M*).

Indeed, the weight of the perfect matching *M *is . According to the double distance formula (see above in this section), this yields *w*(*M*) = 3*n *- *s*(*M*).

Conversely, if *M *is a perfect matching in *G*, its edges between gene extremities define the adjacencies of a genome *M *which satisfies *s*(*M*) = 3*n *- *w*(*M*). This implies that the maximum weight perfect matching solves the guided genome halving problem in the breakpoint distance context.

As is the case for the median problem, it is possible to generalise this statement for an arbitrary number of ordinary outgroup genomes. The phylogenetic problems are open.

Again, we can solve the problem on circular genomes by dropping the *t*_*x *_supplementary vertices in the graph *G*.

### Breakpoint distance, linear case

In this section, *d *= *d*_*BP *_and all genomes must be linear, as is most appropriate for modeling for the eukaryotic nuclear genome. In contrast to the model of the previous section, all the problems concerning at least three genomes are NP-complete.

#### Distance and double distance

The solutions to these problems are the same as in the previous section, where circularity was allowed. In the double distance computation, it is guaranteed that Π ⊕ Π is linear if Π is linear, because if *x *is a telomere in Π, then both *x*1 and *x*2 are telomeres in Π ⊕ Π.

#### Median

Whereas the median is polynomial in the circular and mixed cases, it changes complexity as soon as median genomes are required to be linear. This does not prevent the use of the polynomial algorithm described above as a lower bound, but all biologically relevant median problems seem in fact to be NP-complete.

**Theorem 2**. *The breakpoint median problem for multichromosomal linear genomes is NP-hard*.

*Proof*. We use a reduction from the *2-chromosome breakpoint median*, for which NP-hardness is proved in Lemma 2.

The *2-chromosome breakpoint median *problem takes as input three unichromosomal linear genomes Π_1_, Π_2_, and Π_3 _on a set  of genes, all having the same pair of telomeres. It asks for a linear genome *M *on  with at most two chromosomes, which minimises its median score *s*(*M*) = *d*(Π_1_, *M*) + *d*(Π_2_, *M*) + *d*(Π_3_, *M*). The following lemma states the difficulty of the breakpoint median problem compared to the 2-chromosome breakpoint median problem, and thus, together with Lemma 2, proves Theorem 2.

**Lemma 1**. *Let Π*_1_, Π_2_, Π_3_, *be three unichromosomal linear genomes *Π_1_, *Π*_2_, *and *Π_3 _*all having the same pair of telomeres on the gene set * = {1,...,*n*}, *and k be a positive integer. There exists a genome M on  with at most two linear chromosomes such that s*(*M*) ≤ *k if and only if there exists a multichromosomal linear genome M' on  with s*(*M'*) ≤ *k*.

(⇒): This direction is trivial: simply take *M' *= *M*.

(⇐): Let *M' *be a linear multichromosomal genome satisfying *s*(*M'*) ≤ *k*, that has as few chromosomes as possible. We will prove that *M' *has at most two chromosomes. Suppose *M' *has at least three chromosomes. Then it has at least six telomeres *v*_1_,...,*v*_6_. Among them, it is possible to identify two telomeres (say without loss of generality *v*_1 _and *v*_2_), that belong to different chromosomes and are not telomeres in Π_1_, Π_2_, or Π_3_, because by hypothesis, they all have the same two telomeres. Then the genome constructed from *M' *by adding the adjacency *v*_1_*v*_2 _has at most the same median score as *M' *and fewer chromosomes, contradicting the hypothesis on *M'*. So choosing *M *= *M' *gives a genome with at most two chromosomes such that *s*(*M*) ≤ *k*.   □

We now need to prove the NP-hardness of the *2-chromosome breakpoint median *problem. We use a reduction from the hamiltonian cycle problem for directed graphs with vertex degree at most three, similar to the proof of Bryant [21] for the breakpoint median problem for unichromosomal circular genomes.

**Lemma 2**. *The *2-chromosome breakpoint median *problem is NP-hard*.

*Proof*. Given a directed graph with maximum degree 3, deciding if it has a hamiltonian directed cycle is an NP-complete problem [26]. Let thus *G*_0 _be such a digraph. We will construct an instance of the 2-chromosome breakpoint median problem from *G*_0_.

First, let *G *be the directed graph with vertex set *V *(*G*) = *V *(*G*_0_) ∪ {*xe *: *e *∈ *E*(*G*_0_)} ∪ {*p, q*}, and arc set



Note that *G*_0 _has a hamiltonian cycle if and only if there is a cycle in *G *covering all vertices but *p *and *q*. Given a subset *X *⊆ *E*(*G*) of the arcs of a graph *G*, let *G*_*X *_denote the graph with vertex set *V*(*G*) and arc set *X*.

Construct three subsets *A*, *B*, *C *of arcs of *G *such that every arc of *G *belongs to exactly one of *A*, *B*, *C*, and *A*, *B*, *C *are either hamiltonian cycles of *G *or sets of vertex disjoint paths in *G*. The procedure is straightforward: for all vertex *v *of *G *which is a vertex of *G*_0_, put all incoming arcs in different subsets, and all outgoing arcs in different subsets. It can be done independently for every vertex because from the construction of *G*, no two vertices of *G*_0 _are neighbors. Eventually put arc *pq *in any subset.

**Figure 4:**
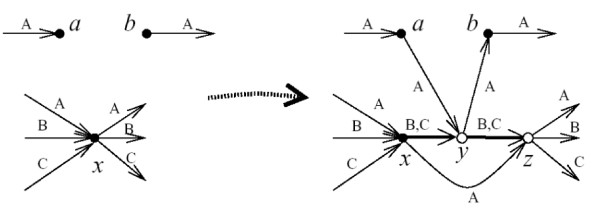
**Reduction of hamiltonian cycle in directed graph to breakpoint median for linear genomes. This figure is redrawn from **[21]. Vertex *a *has no outgoing arc with *X *= *A *in its label set, and *b *has no incoming arc with *A *in its label set. We choose *a, b *such that adding arc *ab *to *G*[*A*] would not give a non-Hamiltonian circuit. We choose an another vertex *x *and insert two new vertices *y *and *z*. The incoming arcs of *x *in the right hand graph are the same as in the left hand graph. The outgoing arcs of *z *are the same as the incoming edges of *x *in the left hand graph. The remaining edges reduce the number of components in *G*[*A*] but leave the same number of components in *G*[*B*] and *G*[*C*].

Now perform a series of modifications of *G *to obtain a graph with three hamiltonian cycles *A*, *B*, and *C*. Along these modifications, we maintain a subset of arcs called *supplementary arcs*, noted *S*. Before any transformation, *S *is empty. The goal is to maintain the property that there exists a cycle covering all vertices of *G *except *p *and *q *and not using supplementary arcs if and only if there is a hamiltonian cycle in *G*_0_. As already remarked, the property is true at the beginning. Choose *X *∈ {*A, B, C*} such that *X *is not a hamiltonian cycle in *G *(it is a set of disjoint paths). Choose two vertices *a *and *b *such that adding the arc *ab *to *G *and *X *would either give a graph in which *X *is a hamiltonian cycle, or a set of disjoint paths with fewer components. Then choose any vertex *x *of *G *different from *a *and *b*. Perform the following transformation illustrated in Figure 4: add two new vertices *y *and *z*. For each arc *xw *of *G*, replace it by the arc *zw*, and add *zw *to *Y *∈ {*A, B, C*} whenever *xw *∈ *Y*. Add arcs *xy*, *yz*, *xz*, *ay*, *yb *to *G*. Add *xz*, *ay*, *yb *to *X*. Also add *xz*, *ay*, *yb *to *S*, and add *xy*, *yz *to all {*A, B, C*}\*X*. Clearly, the property that there exists a cycle covering all vertices of *G *except *p *and *q *and not using supplementary arcs if and only if there is a hamiltonian cycle in *G*_0 _is still true after this transformation.

Repeat this process until *A*, *B *and *C *are all hamiltonian cycles in the resulting graph, which we call *G'*. The *weight *of an arc of *G' *is the number of hamiltonian cycles among *A*, *B *and *C *which contain this arc. Note that *G' *has only weight 1 and 2 arcs.

Let *v *be an arbitrary vertex of *G*, different from *p *and *q*. Let  = *V*(*G'*)\{*v*} ∪ {*v*_1_, *v*_2_} be a set of genes (*v*_1 _and *v*_2 _are two new genes). For every *X *∈ {*A, B, C*}, construct a genome Π_*X *_on  such that *x*^*h*^*y*^*t *^is an adjacency in genome *X *if *xy *is an arc of *X *in *G' *and *x*, *y *are different from *v*; If *xv *and *vy *are the arcs of *X *covering *v*, add the adjacencies  and . This gives three linear unichromosomal genomes on  with the same pair of telomeres ( and ), thus an instance of the *2-chromosome breakpoint median *problem. For any genome *M *on , the *weight *of an adjacency *xy *is the number of genomes, among Π_*A*_, Π_*B *_and Π_*C*_, which contain this adjacency. Adjacencies *u*^*h*^*w*^*t *^in *M *such that *uw *is an arc in *S *are called *supplementary adjacencies*.

Let *λ*_*i *_be the number of arcs of weight *i *in the graph *G'*, for each 0 ≤ *i *≤ 3. For any genome *M *on , note *s*(*M*) = *d*(Π_*A*_, *M*) + *d*(Π_*B*_, *M*) + *d*(Π_*C*_, *M*). The following is inspired by a result from [9] used in [21].

**Claim 4**. *Let n *= |*V *(*G'*)|. *A genome M with N*_*M *_*linear chromosomes on  satisfies s*(*M*) ≥ 2*n *- 1 + *N*_*M *_- *λ*_2_, *where equality holds if and only if M contains all adjacencies of weight 2, and no adjacency of weight 0*.

Indeed, for a genome *M*, denote by *w*(*xy*) the weight of the adjacency *xy*, and *l*_*i *_= |*xy *adjacency of *M*: *w*(*xy*) = *i*|, for each 0 ≤ *i *≤ 3. Let *t*_1 _= 1 if *M *has  as a telomere, and *t*_1 _= 0 otherwise, and *t*_2 _= 1 if *M *has  as a telomere, and *t*_2 _= 0 otherwise. Then we may write . As genome *M *has *N*_*M *_chromosomes, we have *l*_3 _+ *l*_2 _+ *l*_1 _+ *l*_0 _= ||*N*_*M *_and || = *n *+ 1, so . As by construction no arc of *G' *has weight 3, we may write *s*(*M*) ≥ 2*n *- 1 + *N*_*M *_- *λ*_2_. Equality holds if and only if *l*_2 _= *λ*_2_, *l*_0 _= 0, and , that is, if and only if *M *contains all adjacencies of weight at least 2, and no adjacency of weight 0, because *l*_0 _= 0 implies that  and  are telomeres of *M*, thus .

**Claim 5**. *There is a linear genome M on  with at most two chromosomes, with s*(*M*) = 2*n *- 1 + *N*_*M *_- *λ*_2 _*if and only if there is a hamiltonian cycle in G*_0_.

(⇒) Suppose there is a linear genome *M *on  with at most two chromosomes, with *s*(*M*) = 2*n *- 1 + *N*_*M *_- *λ*_2_. This implies by Claim 4 that *M *contains all adjacencies of weight 2, no adjacency of weight 0, and that  and  are telomeres of *M*. From the construction of *G'*, *M *cannot contain any supplementary adjacency, since the extremities of supplementary adjacencies all are also extremities of weight two adjacencies, which are all contained in *M*. Note that in *G'*, paths between vertices *p *and *q *to other vertices of the graph necessarily contain supplementary arcs. This yields that *M *has two chromosomes, one containing gene extremities from *p *and *q*, and the other containing the gene extremities from the other vertices, with telomeres  and . Let *H *⊆ *E*(*G'*) contain the arcs *xy *such that *x*^*h*^*y*^*t *^is an adjacency in *M*, plus the arcs *xv *and *vy *for adjacencies of type  and  in *M*.

*H *∩ *E*(*G*_0_) yields a hamiltonian cycle in *G*_0_.

(⇐) Suppose there is a hamiltonian cycle in *G*_0_. Then there is a cycle *H *covering all vertices of *G' *except *p *and *q*. Construct genome *M *on  by adding (1) adjacencies *x*^*h*^*y*^*t *^whenever *xy *is in *H *and *x, y *are different from *v *(2) adjacencies  and  whenever *xv *or *vy *are arcs of *H*, (3) all weight two adjacencies, and (4) the adjacency *p*^*t*^*q*^*h*^. It is easy to check that *M *is a genome on , and by construction it contains all weight 2 adjacencies and no weight 0 adjacencies.

This proves that it is NP-complete to decide if a median genome reaches the lower bound of Claim 4 for its score, thus computing the minimum score median genome is NP-hard.   □

A byproduct of this proof is the NP-hardness of the 2-chromosome breakpoint median problem. The result implies NP-completeness of the general case, where the genomes in the instance do not necessarily have the same pairs of telomeres. A consequence is that for any fixed *k*, it is also NP-hard to compute a best linear median genome with at most *k *chromosomes.

#### Halving

Surprisingly, this problem has not been treated in the literature. We conjecture it has a polynomial solution, because the halving problem for all other rearrangement distances is polynomial. Constructing a solution is beyond the scope of this paper, and the problem remains open.

#### Guided Halving

This problem is NP-hard, as proved in [27], using the NP-completeness result for the median proved just above in this section.

### DCJ distance, general case

In this section, *d *= *d*_*DCJ*_. Genomes can have several chromosomes, circular or linear. This is the most general context in which the DCJ distance has been explicitly formulated [3]. Genomes rarely contain both circular and linear chromosomes; eukaryote nuclear genomes contain multiple linear chromosomes while prokaryotes generally contain one large circular chromosome, sometimes with additional *plasmids*.

Nevertheless, the simplicity of the computational framework where both genomes may contain both circular and linear chromosomes makes it attractive to mathematical study.

Note that the complexity of the median problem is not established by the work of Caprara [22], who proved the unichromosomal result only. We show the NP-hardness of the multichromosomal case here. Alekseyev and Pevzner [16] mention that the complexity of the double distance problem in the context of circular genomes is open; we show here that it is NP-hard as well.

#### Distance

There is an easy linear solution, both for the distance and the scenario computation [3,12]. We briefly recall the formula for computing the distance, because the underlying principle will be used in our proofs later on in this paper.

**Figure 5:**
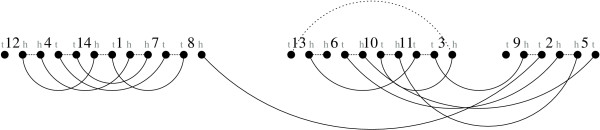
**A Breakpoint Graph**. The breakpoint graph of the genomes Π (see Figure 1) and Γ, given by the union of *C*_1 _= {*T *12^*t*^, 12^*h*^14^*h*^, 14^*t*^7^*h*^, 7^*t*^4^*t*^, 4^*h*^1^*h*^, 1^*t*^8^*t*^, 8^*h*^2^*t*^, 2^*h*^6^*t*^, 6^*h*^*T*} and *C*_2 _= {*T *9^*t*^, 9^*h*^3^*t*^, 3^*h*^10^*t*^, 10^*h*^5^*t*^, 5^*h*^11^*h*^, 11^*t*^13^*h*^, 13^*t*^*T*}. Π-edges are dotted lines, and Γ-edges are plain lines.

The *breakpoint graph *of two genomes Π and Γ on a gene set , denoted by *BP*(Π, Γ), is the graph whose vertex set is the set of extremities of the genes in , where there is an edge between two vertices *x *and *y *if *xy *is an adjacency in either Π (these are Π-edges) or Γ (Γ-edges). Note that we do not invoke any ∘ symbols in the construction of the breakpoint graph. Vertices in this graph have degree zero, one or two, so that the graph is a set of paths (possibly including some with no edges) and cycles. It is also the line-graph of the *adjacency graph*, an alternate representation in [3]. Figure 5 shows an example of a breakpoint graph. Theorem 3 shows how to obtain the distance directly from the graph. The formula is presented in [3] with the cycles and odd paths of the adjacency graph. This corresponds to cycles and even paths of the breakpoint graph, as it is the line-graph of the adjacency graph.

**Theorem 3**. [3]*For two genomes Π and Γ on a gene set  of size n, let c*(Π, Γ) *be the number of cycles of the breakpoint graph BP*(Π, Γ), *and p*(Π, Γ) *be the number of paths with an even number of edges. Then*



Note the similarity to the breakpoint distance formula in the background section on page 5. The number of genes *n *is the same in both formulae, the parameter *c *is related to parameter *a *in the breakpoint formula in that each common adjacency is a cycle of the breakpoint graph (with two parallel edges), and parameter *p *is related to parameter *e*, as each shared telomere is an even path (with no edge) in the breakpoint graph. Although these two measures of genomic distance were derived in different contexts and through different reasoning, their formulae show a remarkably similar form. They differ in that the DCJ formula also counts non-trivial cycles and paths, but for distant genomes, both measures tend to give similar values.

#### Double distance

The NP-completeness proof for the double distance problem follows the principles of Caprara's hardness proof for the median problem in the unichromosomal case [22].

**Theorem 4**. *The DCJ double distance problem is NP-hard for multichromosomal mixed or circular genomes*.

*Proof*. The reduction is from the *breakpoint graph decomposition *(BGD) problem (see [22]). A graph *G *is *bicoloured *if all its edges are coloured either red or blue; it is *balanced *if it has only degree 2 or degree 4 vertices, every vertex is incident to the same number of red and blue edges, and there is no cycle formed by only red or only blue edges. Given a balanced bicoloured graph *G*, the breakpoint graph decomposition problem is to find a partition of the edges of *G *into a maximum number of edge-disjoint cycles, each alternating between red and blue edges. Caprara [22] first proved the NP-hardness of this problem, and Berman and Karpinski [28] extended this by proving APX-hardness.

**Figure 6:**
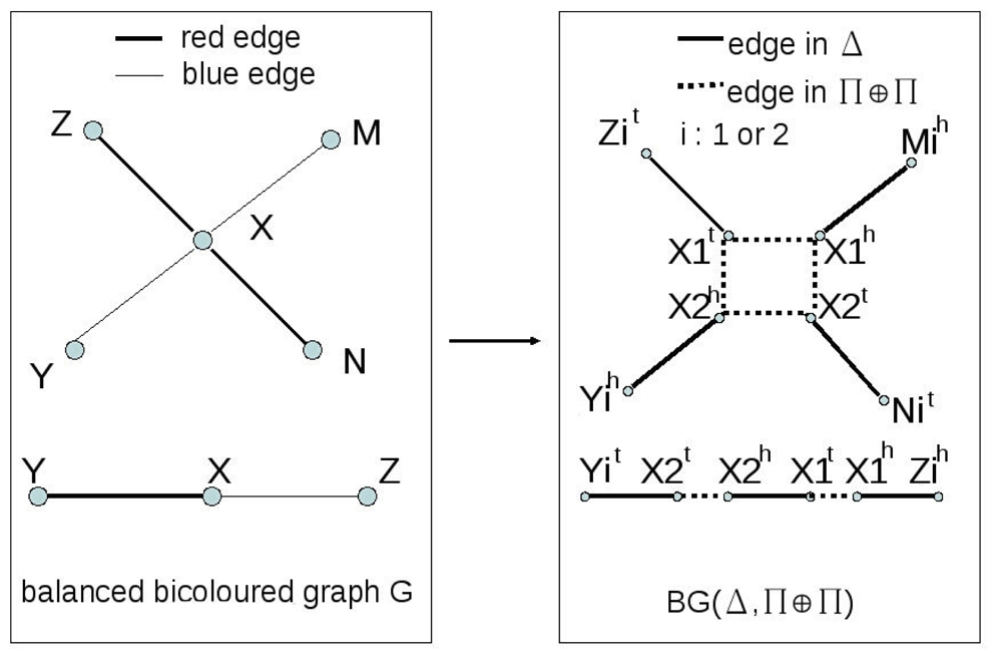
**Reduction of BGD to DCJ double distance problem**. The left hand graph is the balanced bicoloured graph *G*, and the right hand graph represents the adjacencies of the duplicated genomes Δ and Π ⊕ Π. In the case of a degree 2 vertex in *G*, the adjacencies of Π ⊕ Π are determined, as one solution gives more cycles. In the case of a degree 4 vertex in *G*, the two possibilities for the adjacencies of Π ⊕ Π are shown (Π ⊕ Π contains either the vertical or horizontal dotted adjacencies).

Let *G *be a balanced bicoloured graph on *n *vertices, defining an instance of the BGD problem. Let *w*2 be the number of degree 2 vertices of *G*, and *w*4 be the number of degree 4 vertices of *G*. Define the gene set  as the vertex set of *G*. Construct an all-duplicates genome Δ and a genome Π on  in the following way, as illustrated in Figure 6. First, for each gene *X *of , let *X*^*t*^*X*^*h *^be an adjacency in Π. Then, for every vertex *X *of *G*, let *X*1^*t*^, *X*1^*h*^, *X*2^*t *^and *X*2^*h*^, be the extremities of the duplicated gene *X*. If *X *has degree two in *G*, add the adjacency *X*1^*t*^*X*2^*h *^in Δ (if *X *has degree four, no adjacency is added at this point). Then for each blue edge *XY *in *G*, choose among *X*1^*h *^and *X*2^*h *^an extremitiy that is not yet involved in an adjacency, and another among *Y*1^*h *^and *Y*2^*h *^(arbitrarily if neither is involved in an adjacency yet). Add an adjacency between the two chosen extremities in Δ. Then for each red edge *XY *in *G*, choose among *X*1^*t *^and *X*2^*t *^an extremitiy that is not yet involved in an adjacency, and another among *Y*1^*t *^and *Y*2^*t *^(arbitrarily if neither is involved in an adjacency yet). Add an adjacency between the two chosen extremities in Δ.

We then have an all-duplicates genome Δ, and a genome Π. Note that Π is composed of *n *circular chromosomes, one for each gene, and that neither Π nor Δ have telomeres.

**Claim 6**. *The maximum number of edge-disjoint alternating cycles in G is equal to *2*n *- *d*(Δ, Π) - *w*2.

(This claim implies the theorem).

We first prove that the maximum number of edge-disjoint alternating cycles in *G *is at least 2*n *- *d*(Δ, Π) - *w*2. Let Π ⊕ Π be the doubled genome such that *d*(Δ, Π ⊕ Π) = *d*(Δ, Π). As no genome has a telomere, by Theorem 3, *d*(Δ, Π ⊕ Π) = 2*n *- *c*(Δ, Π ⊕ Π). Therefore there are *c*(Δ, Π ⊕ Π) edge-disjoint cycles in *BG*(Δ, Π ⊕ Π) alternating between Π ⊕ Π-edges and Δ-edges. Among them, *w*2 cycles are containing only two edges: if a vertex *X *of *G *has degree 2, then Π ⊕ Π has the adjacency *X*1^*t*^*X*2^*h *^and *X*2^*t*^*X*1^*h *^because the other possibility systematically has one cycle less in *BG*(Δ, Π ⊕ Π). The Δ edges of all the other cycles are the edges of an alternating blue-red cycle in *G*. Indeed, every blue edge defines an adjacency in Δ containing two gene heads, and every red edge defines an adjacency containing two gene tails. The Π ⊕ Π-edges all join one tail and one head, so two consecutive Δ-edges in a cycle of *BG*(Δ, Π ⊕ Π) have different colours. This means there are at least 2*n *- *d*(Δ, Π) - *w*2 alternating cycles in *G*.

Conversely, if there are *k *edge-disjoint alternating cycles in *G*, then *d*(Δ, Π) ≤ 2*n *- *k *- *w*2. Indeed, let *C *be any cycle of this partition. For every covered vertex *X *of degree 4 in *G*, let *e *and *f *be two consecutive edges of *C *(say *e *is blue and *f *is red) which are both incident to *X*. If *e *defines an adjacency in Δ which contains *X*2^*h *^and *f *defines an adjacency which contains *X*2^*t*^, choose *X*1^*h*^*X*1^*t *^and *X*2^*h*^*X*2^*t *^as adjacencies for Π ⊕ Π. If *e *defines an adjacency which contains *X*2^*h *^(or *X*1^*h*^) and *f *defines an adjacency which contains *X*1^*t *^(or *X*2^*t*^), choose *X*1^*h*^*X*2^*t *^and *X*2^*h*^*X*1^*t *^as adjacencies for Π ⊕ Π. For vertices of degree 2, always choose *X*1^*h*^*X*2^*t *^and *X*2^*h*^*X*1^*t *^as adjacencies for Π ⊕ Π. In this construction, each red-blue alternating cycle in *G *is a Π ⊕ Π-Δ alternating cycle in *BP*(Π ⊕ Π, Δ) that has at least *k *cycles. And there are *w*2 additional length 2 cycles at each degree 2 vertex. So *d*(Δ, Π) ≤ 2*n *- *k *- *w*2.   □

#### Median

Though effective exact algorithms [29] and heuristics [30,31] are available, we have:

**Theorem 5**. *The DCJ median problem for multichromosomal genomes is NP-hard, even for circular genomes*.

*Proof*. We use a reduction from the breakpoint graph decomposition defined in the proof of Theorem 4, in a way very similar to part of Caprara's proof [22] for the unichromosomal case.

Let *G *be a balanced bicoloured graph on *n *vertices. Define the gene set  as a set containing one gene *X *for every degree 2 vertex of *G*, and two genes *X *and *Y *for every degree 4 vertex of *G*.

**Figure 7:**
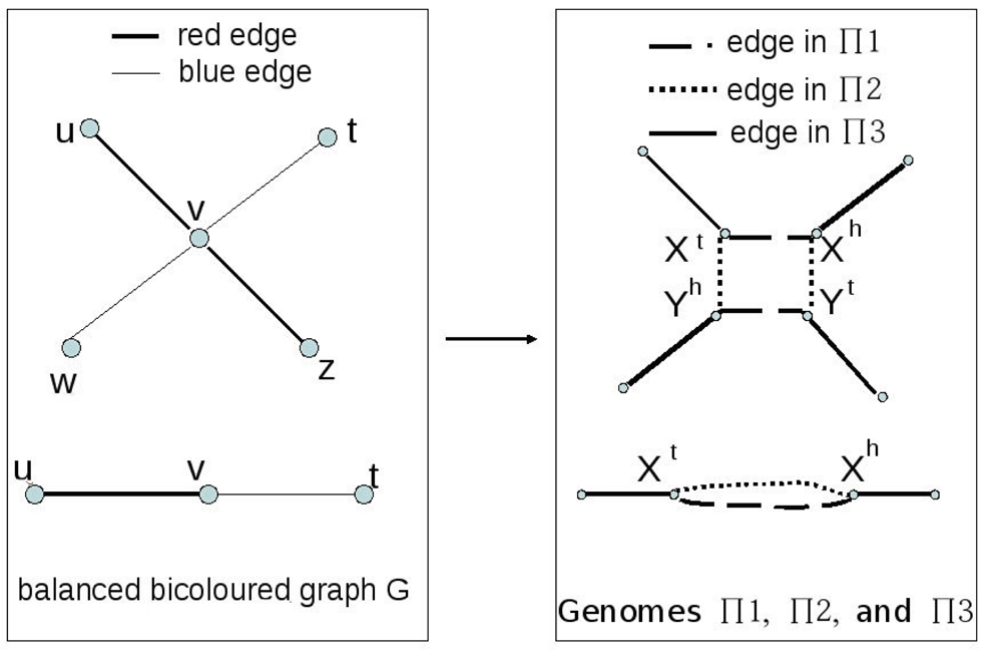
**Reduction of BGD to DCJ median problem**. The left hand graph is the balanced bicoloured graph *G*, and the right hand graph represents the adjacencies of the genomes Π_1_, Π_2 _and Π_3_. Again, in the case of a degree 2 vertex in *G*, the choice for *M *is determined. But in the case of a degree 4 vertex in *G*, either the edges of Π_1 _or Π_2 _can be chosen for the median *M*.

Then construct the genomes Π_1_, Π_2_, Π_3 _in the following way, which is similar to the transformation in [22], as illustrated in Figure 7.

For each degree 4 vertex *v *of *G*, add the two adjacencies *X*^*t*^*X*^*h *^and *Y*^*t*^*Y*^*h *^to Π_1_, and the two adjacencies *X*^*t*^*Y*^*h *^and *Y*^*t*^*X*^*h *^to Π_2_.

Now fo every blue edge *vw *in *G*, add to Π_3 _an adjacency between heads of genes contructed from *v *and *w *(choose one duplicated gene head arbitrarily if *v *or *w *have degree 4). And for every red edge *vu *in *G*, add to Π_3 _an adjacency between tails of genes contructed from *v *and *u *(choose one duplicated gene tail arbitrarily if *v *or *u *have degree 4).

It is easy to see that Π_1_, Π_2_, and Π_3 _define genomes on the set of genes , and they have no telomeres. Let *w*2 be the number of degree 2 vertices of *G*, and *w*4 be the number of degree 4 vertices of *G*.

**Claim 7**. *There exists a genome M on  such that d*(*M*, Π_1_) + *d*(*M*, Π_2_) + *d*(*M*, Π_3_) ≤ *w*2 + 3*w*4* - k if and only if there exists at least k edge-disjoint alternating cycles in G*.

(This claim implies the theorem.)

(⇐): Suppose there are *k *edge-disjoint alternating cycles in *G*. We will construct a median genome *M *such that *d*(Π_1_, *M*) + *d*(Π_2_, *M*) + *d*(Π_3_, *M*) = *w*2 + 2*w*4 - *k*. First, for each degree 2 vertex *v *of *G*, let *X*^*t*^*X*^*h *^be in *M*. Then, let *v *be a degree 4 vertex in *G*, and *vw *be a blue edge incident to *v*. In an alternating cycle, edge *vw *is consecutive with a red edge, say *uv*. To *vw *is associated a constructed Π_3 _adjacency, say *X*^*h*^*W*^*h*^. Then either the Π_3 _adjacency associated to *uv *contains the gene extremity *X*^*t*^, or it contains the extremity *Y*^*t*^. In the first case, let *X*^*h*^*X*^*t *^and *Y*^*h*^*Y*^*t *^be in *M*, and in the second case, let *X*^*h*^*Y*^*t *^and *X*^*t*^*Y *^*h *^be in *M*. The matching *M *defines the adjacencies of a circular genome on , that we also call *M*. There are *w*2 + 2*w*4 genes, so by Theorem 3, *d*(Π_1_, *M*) + *d*(Π_2_, *M*) + *d*(Π_3_, *M*) = 3(*w*2 + 2*w*4) - (*c*(Π_1_, *M*) + *c*(Π_2_, *M*) + *c*(Π_3_, *M*)). By construction, we have *c*(*M*, Π_1_) + *c*(*M*, Π_2_) = 2*w*2 + 3*w*4, and *c*(*M*, Π_3_) = *k*, so *d*(Π_1_, *M*) + *d*(Π_2_, *M*) + *d*(Π_3_, *M*) = *w*2 + 3*w*4 - *k*.

(⇒): Suppose *M *is a genome such that *d*(Π_1_, *M*) + *d*(Π_2_, *M*) + *d*(Π_3_, *M*) ≤ *w*2 + 3*w*4 - *k*. Suppose *M *is chosen such that *d*(Π_1_, *M*) + *d*(Π_2_, *M*) + *d*(Π_3_, *M*) is minimum, and among all such genomes, choose *M *with a maximum number of edges parallel to Π_1_-edges or Π_2_-edges. A circular genome is said to be *canonical *if it only has adjacencies that belong to Π_1 _or Π_2_. We will prove that *M *is canonical.

**Subclaim**. *M *is canonical.

Suppose *M *is not canonical. Suppose first that there is a degree 2 vertex *v *in *G*, such that *M *does not contain the adjacency *X*^*t*^*X*^*h*^. Suppose *M *contains adjacencies *X*^*t*^*a *and *X*^*h*^*b*, where *a *and *b *are gene extremities or ∘ symbols if *X*^*t *^or *X*^*h *^is a telomere in *M*. Then replace *X*^*t*^*a *and *X*^*h*^*b *by *X*^*h*^*X*^*t *^and *ab *(simply *X*^*h*^*X*^*t *^if both *X*^*t *^and *X*^*h *^are telomeres in *M*). By this operation, *c*(*M*, Π_1_) and *c*(*M*, Π_2_) both increase by at least 1, and *c*(*M*, Π_3_) decreases by at most 1, so *d*(Π_1_, *M*) + *d*(Π_2_, *M*) + *d*(Π_3_, *M*) decreases by one, contradicting the hypothesis.

Now suppose that there is a degree 4 vertex in *G*, such that *M *does not contain any of the adjacencies *X*^*h*^*X*^*t*^, *Y*^*h*^*Y*^*t*^, *X*^*h*^*Y*^*t*^, *Y*^*h*^*X*^*t*^. Say it contains adjacencies *X*^*h*^*a*, *X*^*t*^*b*, *Y *^*h*^*c*, *Y*^*t*^*d*, where *a, b, c, d *may be null symbols if any of *X*^*h*^, *X*^*t*^, *Y *^*h*^, *Y*^*t *^is a telomere in *M*. Then replace *X*^*h*^*a*, *X*^*t*^*b*, *Y*^*h*^*c*, *Y*^*t*^*d *by *X*^*h*^*X*^*t*^, *Y*^*h*^*Y *^*t*^, and either *ab*, *cd*, or *ac*, *bd*, or *ad*, *bc*, according to the combination that creates the largest number of cycles in *BP*(*M*, Π_3_). Suppose now that *M *contains only one among the adjacencies *X*^*h*^*X*^*t*^, *Y*^*h*^*Y *^*t*^, *X*^*h*^*Y*^*t*^, *Y*^*h*^*X*^*t*^, say *X*^*h*^*X*^*t*^, and *M *has adjacencies *Y*^*t*^*b *and *Y *^*h*^*c*. Then replace edges *Y*^*t*^*b *and *Y *^*h*^*c *by *Y*^*h*^*Y*^*t *^and *bc*. All these operations decrease *d*(Π_1_, *M*) + *d*(Π_2_, *M*) + *d*(Π_3_, *M*) or maintain it constant, while increasing the number of edges parallel to Π_1 _and Π_2_, contradicting the hypothesis. So the subclaim is proved.

Now, since *M *is canonical, there are *c*(Π_3_, *M*) edge-disjoint alternating cycles in *G*, since an adjacency of *M *always joins a head and a tail, so the corresponding edge in *G *is adjacent to one red edge at one of its vertices and one blue edge at the other. By Theorem 3, *c*(Π_3_, *M*) = 3(*w*2 + 2*w*4) - (*d*(Π_1_, *M*) + *d*(Π_2_, *M*) + *d*(Π_3_, *M*) + *c*(Π_1_, *M*) + *c*(Π_2_, *M*)) and, by hypothesis, *c*(Π_3_, *M*) ≥ 3(*w*2 + 2*w*4) - (*w*2 + 2*w*4 - *k *+ 2*w*2 + 3*w*4), that is, *c*(Π_3_, *M*) ≥ *k*, which proves the claim.   □

#### Halving

This problem has a polynomial solution, as recently stated for unichromosomal genomes by [16] and in the general case by [4,5]. All these algorithms are simplified versions of the algorithm by El-Mabrouk and Sankoff [32], developed for the RT rearrangement distance, which allows reversals, translocations, fusions and fissions, but not the other DCJ operations.

#### Guided Halving

**Theorem 6**. *The DCJ guided halving problem is NP-complete for multichromosomal genomes*.

*Proof*. Again, we use a reduction of the breakpoint graph decomposition problem, as in the proofs of Theorems 4 and 5.

**Figure 8:**
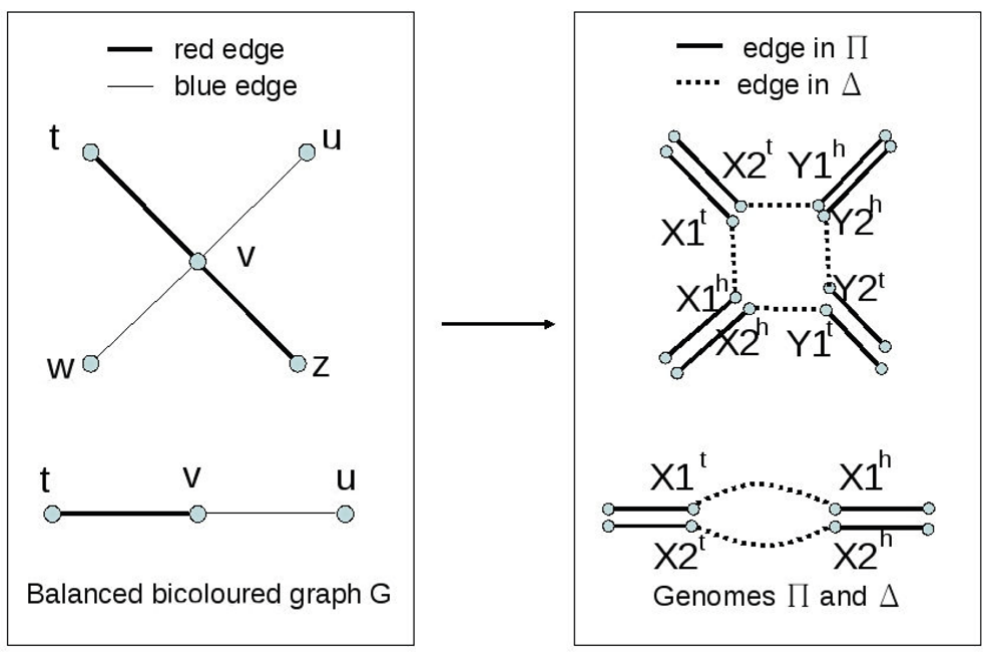
**Reduction of BGD to DCJ guided halving problem**. The left hand graph is the balanced bicoloured graph *G*, and the right hand graph represents the adjacencies of the genomes Δ and Π. Adjacencies of Π are doubled in the drawing to be presented with the doubled genes.

Let *G *be a balanced bicoloured graph on *n *vertices. Define the gene set  as a set containing one gene *X *for every degree 2 vertex of *G*, and two genes *X *and *Y *for every degree 4 vertex of *G*. From *G*, we define one genome Π and one all-duplicates genome Δ on  as illustrated in Figure 8.

For every degree 2 vertex *v *of *G*, let *X *be the corresponding gene in  and *X*^*t *^and *X*^*h *^its extremities. For every degree 4 vertex *v *of *G*, let *X *and *Y *be the two genes in , and *X*^*t *^and *X*^*h*^, *Y *^*t *^and *Y*^*h *^their extremities. For every blue edge *uv *in *G*, construct an adjacency in Π between the heads of the genes constructed from *u *and *v*, choosing arbitrarily between the heads of vertices *X *and *Y *if *u *or *v *have degree 4, in such a way that no two adjacencies share an extremity (choose a different head for the two blue edges incident to a degree 4 vertex). For every red edge *tv *in *G*, construct an adjacency in Π between the tails of the genes constructed from *t *and *v*, again choosing arbitrarily between the tails of vertices *X *and *Y *if *t *or *v *have degree 4. This defines a genome Π on  that has no telomere.

Now, define the genome Δ in the following way. For each degree 2 vertex of *G*, Δ contains the adjacencies *X*1^*h*^*X*1^*t *^and *X*2^*h*^*X*2^*t *^defined by the extremities of the two copies of gene *X*. For each degree 4 vertex in *G*, Δ contains the adjacencies *X*1^*h*^*X*1^*t*^, *Y*2^*h*^*Y*2^*t*^, *X*2^*h*^*Y*1^*t*^, and *X*2^*t*^*Y*1^*h *^defined by the extremities of the two copies of the two genes *X *and *Y*. This defines an all-duplicates genome Δ on  that has no telomere. Let *w*2 be the number of degree 2 vertices of *G*, and *w*4 be the number of degree 4 vertices of *G*.

**Claim**. There exists a genome *M *such that *d*(*M*, Π) + *d*(*M*, Δ) ≤ *w*2 + 3*w*4 - *k *if and only if there exists at least *k *edge-disjoint alternating cycles in *G*. (This claim implies the theorem.)

(⇐): Suppose there are *k *edge-disjoint alternating cycles in *G*. We will construct a genome *M *and a doubled genome *M *⊕ *M *such that *d*(*M*, Π) + *d*(*M*, Δ) ≤ *w*2 + 3*w*4 - *k*. First, for each degree 2 vertex *v *of *G*, let *X*^*t*^*X*^*h *^be in *M*, and *M *⊕ *M *be constructed so that *X*1^*t*^*X*1^*h *^and *X*2^*t*^*X*2^*h *^are in *M *⊕ *M*. Then, for every vertex *v *of degree 4 of *G*, either the blue edge incident to *X*^*h *^is followed by the red edge incident to *X*^*t *^in one alternating cycle, or it is followed by the red edge incident to *Y*^*t*^. In the first case, let *X*^*h*^*X*^*t *^and *Y *^*h*^*Y*^*t *^be in *M*, and *X*1^*h*^*X*1^*t*^, *X*2^*h*^*X*2^*t*^, *Y*1^*h*^*Y*1^*t*^, *Y*2^*h*^*Y*2^*t *^be in *M *⊕*M*. In the second case, let *X*^*h*^*Y*^*t *^and *X*^*t*^*Y*^*h *^be in *M*, and *X*2^*h*^*Y*1^*t*^, *X*1^*h*^*Y*2^*t*^, *X*1^*t*^*Y*2^*h*^, *X*2^*t*^*Y*1^*h *^be in *M *⊕ *M*.

The matching *M *defines a genome without telomeres, and *M *⊕ *M *is one of its doubled genomes. There are *w*2 + 2*w*4 genes, so by Theorem 3, *d*(Π, *M*) = *w*2 + 2*w*4 - *c*(Π, *M*) = *w*2 + 2*w*4 - *k*; and *d*(Δ, *M *⊕ *M*) = 2(*w*2 + 2*w*4) - (2 × *w*2 + 3 × *w*4). So *d*(Π, *M*) + *d*(Δ, *M*) = *w*2 + 3*w*4 - *k*.

(⇒): Suppose *M *is a genome such that *d*(Π, *M*) + *d*(Δ, *M*) ≤ *w*2 + 3*w*4 - *k*. Suppose *M *is chosen such that *d*(Π, *M*) + *d*(Δ, *M*) is minimum, and among all such genomes, choose *M *with a maximum number of edges of type *X*^*t*^*X*^*h *^for a degree 2 vertex in *G*, or *X*^*h*^*X*^*t*^, *Y*^*h*^*Y*^*t*^, *X*^*h*^*Y*^*t *^and *X*^*t*^*Y*^*h *^for degree 4 vertices of *G*. A genome is said to be *canonical *if it has no telomeres and has only edges of type *X*^*t*^*X*^*h *^for a degree 2 vertex in *G*, or *X*^*h*^*X*^*t*^, *Y*^*h*^*Y*^*t*^, *X*^*h*^*Y*^*t *^and *X*^*t*^*Y*^*h *^for degree 4 vertices of *G*. We will prove that *M *is canonical.

**Subclaim**. *M *is canonical.

Suppose *M *is not canonical. Suppose first that there is a degree 2 vertex *v *in *G*, such that *M *does not contain the edge *X*^*t*^*X*^*h*^. Suppose *M *contains edges *X*^*t*^*u *and *X*^*h*^*v*, where *u *and *v *are gene extremities or ∘ symbols if *X*^*t *^or *X*^*h *^is a telomere in *M*. Then replace *X*^*t*^*u *and *X*^*h*^*v *by *X*^*h*^*X*^*t *^and *uv *(simply *X*^*h*^*X*^*t *^is both *X*^*t *^and *X*^*h *^are telomeres in *M*). By this operation, *c*(*M*, Π) decreases by at most one, while defining *M *⊕ *M *as containing *X*1^*h*^*X*1^*t *^and *X*2^*h*^*X*2^*t *^makes *c*(*M *⊕ *M*, Δ) increase by at least 2. This contradicts the hypothesis.

Now suppose that there is a degree 4 vertex in *G*, such that *M *does not contain any of the edges *X*^*h*^*X*^*t*^, *Y*^*h*^*Y*^*t*^, *X*^*h*^*Y*^*t*^, *Y*^*h*^*X*^*t*^. Say it contains edges *X*^*h*^*t*, *X*^*t*^*u*, *Y*^*h*^*v*, *Y*^*t*^*w*, where *t, u, v, w *may be null symbols if any of *X*^*h*^, *X*^*t*^, *Y*^*h*^, *Y*^*t *^is a telomere in *M*. Then replace *X*^*h*^*t*, *X*^*t*^*u*, *Y*^*h*^*v*, *Y*^*t*^*w *by *X*^*h*^, *X*^*t*^, *Y*^*h*^*Y*^*t*^, *tu*, *vw *or *X*^*h*^*Y*^*t*^, *tw*, *X*^*t*^*Y*^*h*^, *uv*, depending on the cycles in *c*(*M *⊕ *M*, Δ).

Suppose now that *M *contains only one among the edges *X*^*h*^*X*^*t*^, *Y*^*h*^*Y*^*t*^, *X*^*h*^*Y*^*t*^, *Y*^*h*^*X*^*t *^say *X*^*h*^*X*^*t*^, and *M *has edges *Y*^*t*^*u *and *Y*^*h*^*v*. Then replace edges *Y*^*t*^*u *and *Y*^*h*^*v *by *Y*^*h*^*Y*^*t *^and *uv*. All these operations decrease *d*(Π, *M*) + *d*(Δ, *M*) or maintain it constant, while increasing the number of edges of type *X*^*t*^*X*^*h *^for a degree 2 vertex in *G*, or *X*^*h*^*X*^*t*^, *Y*^*h*^*Y*^*t*^, *X*^*h*^*Y *^*t *^and *X*^*t*^*Y*^*h *^for degree 4 vertices of *G*, contradicting the hypothesis. At the end of this process, *M *is canonical, so the subclaim is proved.

Now, since *M *is canonical, there are *c*(Π, *M*) edge-disjoint alternating cycles in *G*, since an edge of *M *always joins a head and a tail, so that it is adjacent to one red and one blue edge. By Theorem 3, *c*(Π, *M*) = 3(*w*2 + 2*w*4) - (*d*(Π, *M*) + *d*(Δ, *M *⊕ *M*) + *c*(Δ, *M *⊕ *M*)), and by hypothesis, *c*(Π_3_, *M*) ≥ 3(*w*2 + 2*w*4) - (*w*2 + 3*w*4 - *k *+ 2*w*2 + 3*w*4)), that is, *c*(Π_3_, *M*) ≥ *k*, which proves the claim.   □

### DCJ distance, linear chromosomes

In the original formulation of the DCJ distance [12], it was shown that there is a solution where each excision of a circular intermediate could be followed directly by its reinsertion. Thus the median and halving problems can be stated in terms of exclusively linear chromosomes in both the data genomes and the reconstructed ancestor. They all remain open.

### Reversal/Translocation distance

Hannenhalli and Pevzner proposed a polynomial-time algorithm for calculating *d*_*RT *_(Π, Γ) for two genomes Π and Γ [17], after solving the problem for unichromsomal genomes [39]. This was reformulated in [33], minor corrections were added in [34] and [35], and Bergeron *et al*. simplified the formula [14] and investigated the relations between *d*_*RT *_and *d*_*DCJ*_.

A polynomial time genome halving algorithm was given in [32]. Though the constrained DCJ distance in the previous section is arguably just as realistic, because of the long history of *d*_*RT*_, effective heuristics for RT have been developed and applied for the double distance [18,36], median [31,37] and guided halving problems [18,19,36], but their complexities remain open questions. Note that [38] gives an NP-completeness result on a problem which slightly generalizes the reversal double-distance probem on unichromosomal genomes.

## Discussion and conclusion

Table 1 summarises the current knowledge of the complexity of the five genome rearrangement problems, including the new results in this paper. Note that all the results on general multichromosomal genome (that is, circular or linear) also hold for exclusively circular genomes, as the polynomial algorithms can always provide a circular solution to a circular instance, and all NP-completeness proofs are constructed with circular chromosomes.

**Table 1:** Results summary.

problem context: distance, #chr, linear, circular or mixed	distance	halving	double distance	median	guided halving
breakpoint unichr, circular or linear	P	open	open	NP [20,21]	open
breakpoint multichr, circular and mixed	P new	P new	P new	P new	P new
breakpoint multichr, linear	P new	open P?	P new	NP new	NP [27]

DCJ unichr, circular or linear	P [3,12]	P [16]	open	NP [22]	open
DCJ multichr, circular and mixed	P [3,12]	P [4,5]	NP new	NP new	NP new
DCJ multichr, linear	P [12]	open	open	open NP?	open NP?

RT unichr	P [39]	open	open	NP [22]	open
RT multichr	P [17,33-35]	P [32]	open NP?	open NP?	open NP?

Status of complexity questions for five problems related to ancestral genome reconstruction, for eight genomic distances in the unichromosomal and multichromosomal contexts. Note that unichromosomal problems require that both input and output genomes be unichromosomal, so all problems involving doubled genomes are computationally defined in the circular case, when the doubled genome consists in a single circular chromosome composed of two successive occurences of the ordinary genome. Other versions of the halving problem are less restrictive [5,16,32]. P and NP stand for polynomial and NP-hard, respectively, and when followed by ?, represent our conjectures.

## Authors' contributions

ET, CZ and DS have elaborated the definition and conjectures, proved the results and written the paper.

## References

[B1] Fertin G, Labarre A, Rusu I, Tannier E, Vialette S (2009). Combinatorics of Genome Rearrangements.

[B2] Tannier E, Zheng C, Sankoff D (2008). Multichromosomal genome median and halving problems. Algorithms in Bioinformatics, proceedings of WABI'08, of Lecture Notes in Bioinformatics.

[B3] Bergeron A, Mixtacki J, Stoye J (2006). A unifying view of genome rearrangements. Algorithms in Bioinformatics, proceedings of WABI'06, of Lecture Notes in Computer Science.

[B4] Mixtacki J (2008). Genome Halving under DCJ revisited. Proceedings of COCOON'08, Lecture Notes in Computer Science.

[B5] Warren R, Sankoff D (2008). Genome halving with double cut and join. Proceedings of the 6th Asia-Pacific Bioinformatics Conference, of Advances in Bioinformatics and Computational Biology.

[B6] Aury J, Jaillon O, Duret L, Noel B, Jubin C, Porcel B, Ségurens B, Daubin V, Anthouard V, Aiach N, Arnaiz O, Billaut A, Beisson J, Blanc I, Bouhouche K, Câmara F, Duharcourt S, Guigó R, Gogendeau D, Katinka M, Keller A, Kissmehl R, Klotz C, Koll F, Mouël AL, Lepère G, Malinsky S, Nowacki M, Nowak J, Plattner H, Poulain J, Ruiz F, Serrano V, Zagulski M, Dessen P, Bétermier M, Weissenbach J, Scarpelli C, Schächter V, Sperling L, Meyer E, Cohen J, Wincker P (2006). Global trends of whole-genome duplications revealed by the ciliate Paramecium tetraurelia. Nature.

[B7] Otto S, Whitton J (2000). Polyploid incidence and evolution. Annual Review of Genetics.

[B8] Watterson G, Ewens W, Hall T, Morgan A (1982). The chromosome inversion problem. Journal of Theoretical Biology.

[B9] Sankoff D, Blanchette M (1997). The median problem for breakpoints in comparative genomics. Proceedings of the Third International Computing and Combinatorics Conference COCOON'97, of Lecture Notes in Computer Science.

[B10] Pevzner P, Tesler G (2003). Transforming men into mice: the Nadeau-Taylor chromosomal breakage model revisited. Proceedings of the seventh annual international conference on Research in computational molecular biology RECOMB'03.

[B11] Bergeron A, Mixtacki J, Stoye J (2008). On computing the breakpoint reuse rate in rearrangement scenarios. Proceedings of Recomb Workshop on Comparative Genomics, Lecture Notes in Bioinformatics.

[B12] Yancopoulos S, Attie O, Friedberg R (2005). Efficient sorting of genomic permutations by translocation, inversion and block interchange. Bioinformatics.

[B13] Lin Y, Lu C, Chang HY, Tang C (2005). An efficient algorithm for sorting by block-interchange and its application to the evolution of vibrio species. Journal of Computational Biology.

[B14] Bergeron A, Mixtacki J, Stoye J (2008). HP distance via Double Cut and Join distance. Combinatorial Pattern Matching, proceedings of CPM'08, of Lecture Notes in Computer Science.

[B15] Lin YC, Lu CL, Liu YC, Tang CY (2006). SPRING: a tool for the analysis of genome rearrangement using reversals and block-interchanges. Nucleic Acids Res.

[B16] Alekseyev M, Pevzner PA (2008). Colored de Bruijn graphs and the genome halving problem. IEEE/ACM Transactions on Computational Biology and Bioinformatics.

[B17] Hannenhalli S, Pevzner P (1995). Transforming men into mice (polynomial algorithm for genomic distance problem). Proceedings of the 36th Annual Symposium on Foundations of Computer Science FOCS'95).

[B18] Zheng C, Zhu Q, Sankoff D (2008). Descendants of whole genome duplication within gene order phylogeny. Journal of Computational Biology.

[B19] Zheng C, Zhu Q, Sankoff D (2006). Genome halving with an outgroup. Evolutionary Bioinformatics.

[B20] Pe'er I, Shamir R (1998). The median problems for breakpoints are NP-complete. Electronic Colloquium on Computational Complexity.

[B21] Bryant D (1998). The complexity of the breakpoint median problem. Tech Rep CRM-2579.

[B22] Caprara A (2003). The reversal median problem. INFORMS Journal on Computing.

[B23] Ohlebusch E, Abouelhoda MI, Hockel K (2007). A linear time algorithm for the inversion median problem in circular bacterial genomes. J of Discrete Algorithms.

[B24] Bernt M, Merkle D, Middendorf M (2008). Solving the Preserving Reversal Median Problem. IEEE/ACM Transactions on Computational Biology and Bioinformatics.

[B25] Lovasz L, Plummer MD (1986). Matching Theory, of Annals of Discrete Mathematics Amsterdam: North Holland.

[B26] Garey MR, Johnson DS (1979). Computers and intractability A guide to the theory of NP-completness.

[B27] Zheng C, Zhu Q, Adam Z, Sankoff D (2008). Guided genome halving: hardness, heuristics and the history of the Hemiascomycetes. Bioinformatics.

[B28] Berman P, Karpinski M (1999). On some tighter inapproximability results. Automata, Languages and Programming, of Lecture Notes In Computer Science.

[B29] Xu W, Sankoff D (2008). Decompositions of Multiple Breakpoint Graphs and Rapid Exact Solutions to the Median Problem. Algorithms in Bioinformatics, proceedings of WABI'08, Lecture Notes in Bioinformatics.

[B30] Adam Z, Sankoff D (2008). The ABCs of MGR with DCJ. Evol Bioinform Online.

[B31] Lenne R, Solnon C, Stützle T, Tannier E, Birattari M (2008). Reactive stochastic local search algorithms for the genomic median problem. Proceedings of EvoCOP'08, of Lecture Notes in Computer Science.

[B32] El-Mabrouk N, Sankoff D (2003). The reconstruction of doubled genomes. SIAM Journal of Computing.

[B33] Tesler G (2002). Efficient algorithms for multichromosomal genome rearrangements. Journal of Computer and System Sciences.

[B34] Ozery-Flato M, Shamir R (2003). Two notes on genome rearrangement. Journal of Bioinformatics and Computational Biology.

[B35] Jean G, Nikolski M (2007). Genome rearrangements: a correct algorithm for optimal capping. Information Processing Letters.

[B36] Zheng C, Wall PK, Leebens-Mack J, de Pamphilis C, Albert V, Sankoff D (2008). The effect of massive gene loss following whole genome duplication on the algorithmic reconstruction of the ancestral Populus diploid. Proceedings of CSB'08.

[B37] Bourque G, Pevzner P (2002). Genome-scale evolution: Reconstructing gene orders in the ancestral species. Genome Research.

[B38] Chen X, Zheng J, Fu Z, Nan P, Zhong Y, T Jiang SL (2005). Assignement of orthologous genes via genome rearrangement. IEEE/ACM Transactions on Computational Biology and Bioinformatics.

[B39] Hannenhalli S, Pevzner P (1999). Transforming Cabbage into Turnip: Polynomial Algorithm for Sorting Signed Permutations by Reversals. Journal of the ACM.

